# A Comparative Analysis of Physical Demands Across Different Small-Sided Game Formats in a South African Premier Soccer League Team

**DOI:** 10.3390/jfmk11040412

**Published:** 2026-10-08

**Authors:** Mduduzi Rhini, Robert Hickner, Rowena Naidoo, Takshita Sookan-Kassie

**Affiliations:** 1 Department of Human Movement Sciences, Faculty of Health Sciences, University of Fort Hare, Dikeni 5700, South Africa; 2 Discipline of Biokinetics, Exercise and Leisure Sciences, School of Health Sciences, University of KwaZulu-Natal, Durban 4041, South Africa; rhickner@fsu.edu (R.H.); naidoor3@ukzn.ac.za (R.N.); sookan@ukzn.ac.za (T.S.-K.); 3 Department of Health, Nutrition and Food Sciences, College of Education, Health, and Human Sciences, Florida State University, Tallahassee, FL 32306, USA

**Keywords:** small-sided games, training load, physical demands

## Abstract

**Background:** Anecdotal evidence shows that small-sided games (SSGs) are the most preferred training method, as they integrate the reality of a match into training. However, it remains unclear which specific format/size best replicates the demands of competitive matches. **Objective:** This study aimed to quantify the physical demands associated with different SSG formats used by a South African Premier Soccer League team. **Methods:** Data were retrospectively collected from players within the same team using PlayerTek (10 Hz) GPS devices. Variables measured included total distance covered, high-intensity running distance, power plays, top-end speed, and distance covered per minute. Small-sided games were categorized by player number: small (5v5), medium (6v6 and 7v7), and large (8v8, 9v9, and 10v10). **Results:** Physical demands varied significantly across SSG formats (*p* = 0.001). Total distance covered was greater in 5v5 and 10v10 formats compared to 6v6 and 7v7. The 10v10 format showed higher high-intensity running distances than all other formats. Similar trends were observed for top-end speed and power plays, although 5v5 games recorded more power plays than 6v6, 7v7, and 9v9 formats. Additionally, 5v5 formats were associated with greater distance covered per minute than 7v7, 9v9, and 10v10 formats. **Conclusions:** Different SSG formats were characterized by distinct physical demands on players. Larger SSGs, particularly 10v10, were associated with greater high-intensity running and maximal-speed actions, whereas 5v5 formats were associated with greater relative work rates and repeated high-intensity efforts. These findings may assist coaches when selecting SSG formats according to specific training objectives.

## 1. Introduction

Soccer is a dynamic, complex, and inherently nondeterministic sport in which players continuously interact with the ball, teammates, opponents, and space. Performance in soccer in multifactorial, relying on the integration of physical, technical, tactical and cognitive abilities [1]. Consequently, training methods that simultaneously develop these performance components are considered particularly effective for preparing players for the demands of competition. One such method is the use of small-sided games (SSGs), which have become increasingly popular in both professional and amateur soccer settings [2]. Small-sided games are modified games played on smaller pitch areas, with a smaller number of players, adapted rules, and task constraints, such as limitations on ball touches or the inclusion or exclusion of goalkeepers and goals [3]. By reproducing key characteristics of competitive match-play while allowing the manipulation of training constraints, SSGs provide an effective and sport-specific means of simultaneously developing technical, tactical, and physical performance capacities. As a result, they have become one of the most extensively researched and widely implemented training methods in soccer over the past two decades [1,4].

Numerous studies have investigated the effectiveness of SSGs as a training modality [5,6,7], with findings suggesting that they can provide physiological and physical stimuli comparable to those elicited by traditional high-intensity running-based training methods [3,8,9]. Despite the growing body of literature, uncertainty remains regarding which SSG format best replicates the physical and physiological demands of competitive match-play. Small-sided games can vary substantially in terms of player numbers, pitch dimensions, and task constraints, all of which influence the intensity and nature of the training stimulus. Research suggests that SSGs involving fewer players, such as 3v3, 4v4, and 5v5 formats, generally promote greater player involvement and more frequent interactions with the ball than larger formats such as 9v9, 10v10, and 11v11 [10]. For example, Dellal et al. [11] reported that high-intensity and very-high-intensity running distances were greater during 2v2 games than during 3v3 and 4v4 formats. The authors attributed these findings to reduced recovery opportunities and a greater frequency of high-intensity actions in games with fewer players. Similarly, Rampinini et al. [12] demonstrated that smaller SSG formats elicited higher exercise intensities than medium- and large-sided games.

Conversely, more recent evidence has suggested that larger SSG formats may be more effective in reproducing the locomotor demands of competitive matches. Beato et al. [5] found that although small and large SSGs elicited similar internal loads, as measured by ratings of perceived exertion, larger formats resulted in greater relative distance covered, high-speed running distance, and sprinting distance. The authors concluded that larger SSGs may better replicate the external load demands of match-play than smaller formats. The discrepancies observed across studies may be explained by differences in task-configuration variables, including pitch dimensions, player numbers, and area per player (ApP—expressed as m^2^.player^−1^). These variables are known to substantially influence the physiological and physical responses elicited during SSGs and should therefore be carefully controlled when designing training interventions [2,13]. Previous research has demonstrated that increasing pitch size results in greater total distance covered, as well as increased high-intensity running and sprinting distances [14,15]. Larger playing areas provide players with more opportunities to perform longer runs and achieve higher movement velocities than smaller pitches. Furthermore, Riboli et al. [1] reported a strong relationship between ApP and the ability of SSGs to replicate the physical demands observed during competitive matches.

Given the widespread use of SSGs in soccer training, understanding the physical demands associated with different game formats is essential for coaches seeking to prescribe training activities that align with specific performance objectives. Although several studies have examined the effects of different SSG formats, research within the South African professional soccer context remains limited. Differences in playing style, training practices, competitive demands, and environmental conditions may influence the physical responses of players and limit the direct application of findings from international studies. Therefore, the aim of this study was to compare the physical demands associated with small-, medium-, and large-sided game formats employed by a South African Premier Soccer League team. It was hypothesised that significant differences in physical performance variables would exist between SSG formats, reflecting the influence of player numbers and game configuration on training load.

## 2. Materials and Methods

### 2.1. Population

This study employed an observational, descriptive design to examine the physical demands associated with different SSGs formats used within a professional soccer environment. Data were collected from 24 professional male players from a South African Premier Soccer League (PSL) team during the 2019/2020 competitive season. Participant characteristics were as follows (mean ± SD): age 25.5 ± 3.8 years, height 174.0 ± 6.0 cm, body mass 69.8 ± 9.9 kg, and body mass index 23.1 ± 2.2 kg·m^−2^.

Data collection was conducted over a 27-week in-season period during regular training sessions. A total of 913 individual GPS files were analyzed across five playing positions: full backs (FBs), centre backs (CBs), central midfielders (CMs), attacking midfielders (AMs), and centre forwards (CFs). A total of 60 SSG sessions were analysed, comprising ten sessions for each format (5v5, 6v6, 7v7, 8v8, 9v9, and 10v10). The selection of SSG formats was determined by the coaching staff according to the objectives of each training session. Consequently, several training variables differed across SSG formats, including pitch dimensions, ApP, goalkeeper inclusion, tactical objectives, game rules, and work-to-rest structure. These variations reflected the coaching staff’s routine training practices and should be considered when interpreting the findings, as the observational design does not permit the independent effects of player number or SSG format to be isolated.

Although all formats incorporated varying combinations of tactical constraints, offside rules, supporting wall players, and goalkeepers, smaller and medium-sized SSGs were predominantly possession-based activities performed without goalkeepers or full-sized goals. In contrast, larger SSGs were generally game-based activities that included goalkeepers and regulation goals. Furthermore, the number of sets and recovery periods varied across training sessions, ranging from three to five sets with three to five recovery intervals.

### 2.2. Performance Variables Measured

Player movement data were collected using PlayerTek global positioning system (GPS) units (PlayerTek, Dundalk, Ireland) operating at a sampling frequency of 10 Hz, with integrated triaxial accelerometers sampling at 100 Hz. Signal quality indicators, including the number of connected satellites and horizontal dilution of precision (HDOP), were not available from the archived GPS dataset and therefore could not be reported. However, previous research has demonstrated the validity and reliability of these devices for monitoring player movement characteristics in team sports [16]. Each player wore the same GPS unit throughout the study period in a manufacturer-provided vest, which positioned the device between the scapulae on the upper back. Devices were activated approximately 15 min before the start of each training session and deactivated immediately following the completion of the session.

The following external load variables were extracted from the PlayerTek platform: total distance covered (km), high-intensity running distance (m; >18 km·h^−1^), power plays (number of actions performed above 20 W·kg^−1^), top-end speed (km·h^−1^), and relative distance covered (m·min^−1^). The threshold of >18 km·h^−1^ was selected because it corresponds to the default speed classification used by the PlayerTek system and has been adopted in previous studies using the same technology [17].

### 2.3. Procedure

Data were extracted retrospectively from the club’s training-load monitoring database. Small-sided games formed a central component of the team’s training methodology, with different formats used according to the objectives of specific training days. During a typical training week, sessions followed a structured weekly periodization model, consisting of a conditioning-focused session on MD-5, technical and tactical training on MD-4, tactical sessions on MD-3 and MD-2 (including set-play preparation on MD-2), and an activation session with final tactical preparations on MD-1.

Conditioning sessions predominantly utilised smaller SSG formats (5v5), whereas technical and tactical sessions frequently incorporated medium-sized SSGs (6v6 and 7v7). Tactical sessions closer to match day generally employed larger SSG formats (8v8, 9v9, and 10v10) on larger pitch areas to facilitate tactical preparation while exposing players to greater running space.

Players completed the club’s standardised warm-up procedures before each training session. Warm-up content was determined by the coaching staff according to the objectives of the respective training session. The team regularly employed a variety of SSG formats throughout the training week. Six formats were included in the analysis: 5v5, 6v6, 7v7, 8v8, 9v9, and 10v10. For analytical purposes, SSG formats were categorized according to player numbers as small-sided games (5v5), medium-sided games (6v6 and 7v7), and large-sided games (8v8, 9v9, and 10v10).

The pitch dimensions used for each format are presented in Table 1. Because pitch dimensions and area per player increased progressively across the SSG formats, the influence of player number cannot be separated from the influence of available playing space. Briefly, 5v5 games were played on pitches measuring 30 × 20 m, while 6v6 and 7v7 formats were conducted on either 30 × 30 m or 40 × 40 m pitches. The 8v8 and 9v9 formats were played on box-to-box dimensions (80 × 50 m), whereas 10v10 games (11v11 including goalkeepers) were played on a full-sized pitch (110 × 65 m).

Accordingly, the present study should be interpreted as describing external-load differences observed across routinely implemented training configurations rather than evaluating the isolated effects of any single SSG design characteristic.

### 2.4. Ethical Considerations

Consent to participate in the study was given by all players after a detailed explanation about the purpose and benefits of the study. In addition, the University of KwaZulu-Natal’s Biomedical Research Ethics Committee approved the study protocol and granted ethical clearance (BE695/18). Before entering the study, all players were informed of the nature and demands of the study and the potential risks. Furthermore, all players gave written, informed consent in accordance with the Helsinki Declaration.

### 2.5. Statistical Analysis

All data were analysed using IBM SPSS Statistics Version 25 (IBM Corp., Chicago, IL, USA). Descriptive statistics are presented as means ± standard deviations (mean ± SD). Although linear mixed-effects models may provide greater flexibility when analyzing repeated observations with unequal sample sizes and nested data structures, repeated-measures ANOVA was selected to compare average external-load responses across the predefined SSG formats. The limitations associated with this analytical approach are acknowledged and are discussed when interpreting the findings. The dependent variables included total distance covered, high-intensity running distance, power plays, relative distance covered, and top-end speed. Where significant main effects were identified, Bonferroni-adjusted post hoc comparisons were performed to determine pairwise differences between SSG formats. Statistical significance was accepted at *p* < 0.05.

## 3. Results

A significant difference (*p* < 0.005) was evident across all formats. Total distance covered was significantly greater during the small SSGs (5v5) and large SSGs (8v8 and 10v10) formats compared with the medium SSGs (6v6 and 7v7). No significant differences were found between the 5v5, 8v8, and 10v10 formats (Table 2).

High-intensity running distance was significantly greater during the 10v10 format than in all other SSG formats. Similarly, the 10v10 format showed the highest top-end speed values, which were significantly greater than those recorded during all other formats, with the exception of 9v9. The 9v9 format also produced significantly higher top-end speed values than the 5v5, 6v6, and 7v7 formats. Furthermore, top-end speed was significantly greater during 5v5 compared with 6v6.

For power plays, significantly higher values were observed during the 5v5 and 10v10 formats compared with the 6v6 and 7v7 formats. The 8v8 format also produced significantly more power plays than the 6v6 format.

Relative distance covered (m·min^−1^) was significantly greater during the 5v5 format compared with the 7v7, 9v9, and 10v10 formats.

The comparison of SSG formats according to game size (small-, medium-, and large-sided games) is presented in Table 3. Significant differences were observed across all physical performance variables (*p* < 0.005). Total distance covered was significantly greater during small-sided games than during medium- and large-sided games. In addition, large-sided games resulted in significantly greater total distance covered than medium-sided games.

High-intensity running distance was significantly greater during both small- and large-sided games compared with medium-sided games. A similar pattern was observed for power plays, with significantly higher values recorded during small- and large-sided games than during medium-sided games.

For top-end speed, large-sided games recorded significantly higher values than both small- and medium-sided games (*p* < 0.005). Finally, relative distance covered (m·min^−1^) was significantly greater during small-sided games than during medium- and large-sided games.

## 4. Discussion

The aim of this study was to quantify and compare the physical demands associated with different SSG formats used by a South African Premier Soccer League team during the in-season period. The results showed that key performance variables; total distance covered, high-intensity running, power plays, top-end speed, and distance covered per minute varied significantly across the different SSG formats.

With regard to total distance covered, no significant differences were observed between the 5v5, 8v8, and 10v10 formats. However, when SSGs were grouped by overall size, small SSGs were associated with significantly greater total distance covered compared to medium- and large-sided formats. These findings partially contrast with the existing literature, which generally suggests that larger SSGs are more likely to result in greater total distance covered than small and medium SSGs [4,13,18]. This discrepancy may reflect the relative influence of pitch dimensions. Previous research suggests that a higher ApP is more likely to result in increased total distance covered compared to smaller ApP configurations [15]. However, this trend was not observed in the present study. Instead, greater total distance was recorded in smaller pitch formats with fewer players.

A plausible explanation for this finding lies in the contextual application of the SSG formats within the training program. In the present study, small SSGs (5v5) were predominantly implemented during conditioning or loading sessions, whereas larger SSGs (10v10) were primarily used during tactical sessions. This contextual difference likely contributed to the observed outcomes and is further supported by the significantly higher relative values (e.g., distance per minute) recorded during small SSGs compared to medium- and large-sided formats. While small SSGs were characterised by greater total distance covered and higher relative intensity (e.g., distance per minute), a contrasting pattern emerged when examining high-intensity actions. Specifically, large SSGs demonstrated a clear advantage in high-intensity running demands compared to small SSGs, with the exception of power plays, where no significant differences were observed. The results indicate that large SSGs (10v10) were associated with greater high-intensity running distances and higher top-end speed values compared to all other formats, including small SSGs (5v5).

This finding may reflect the influence of pitch dimensions and the greater ApP available in the larger SSG formats. Larger pitch sizes provide greater space, thereby affording players more opportunities and, in some cases, imposing greater demands to reach and sustain higher running speeds, both in and out of possession [13,15]. In support of this, the previous literature has demonstrated a strong relationship between ApP and high-intensity locomotor activity, as well as metabolic power output [1]. Specifically, higher ApP values are associated with increased high-intensity running and elevated physiological responses compared to smaller ApP configurations.

Empirical evidence further substantiates these observations. For instance, Hodgson et al. [9] reported significantly greater high-intensity running, as well as increased acceleration and deceleration demands, on larger pitches compared to smaller ones. Similarly, Clemente et al. [19] found that larger playing areas resulted in significantly higher volumes of high-intensity running, more frequent sprinting actions, and higher top-end speeds. Collectively, these findings align with the present study, reinforcing the notion that larger pitch formats are associated with greater high-intensity demands than small- and medium-SSGs. Importantly, this pattern persists despite the contextual differences in training objectives observed in the present study, where small SSGs were primarily used for conditioning and larger SSGs for tactical purposes. These findings are consistent with previous literature suggesting that larger playing areas may facilitate greater opportunities for high-intensity locomotor actions. However, because pitch size, ApP, goalkeeper inclusion, and tactical objectives varied concurrently in the present study, the independent influence of pitch size cannot be established.

Taken together, the contrasting responses observed between total distance and high-intensity actions highlight the multifactorial nature of SSG design. Several explanations have been proposed in the literature to account for such variability, including methodological differences, task constraints, the presence of goalkeepers, tactical roles, and coaching influence [6,20]. For instance, the inclusion of goalkeepers has been shown to reduce both total distance covered and overall intensity, as their presence facilitates improved defensive organization and positional structure, thereby limiting open-play movement demands [7].

In addition, task constraints such as ball-touch restrictions can substantially influence physical outputs. Gimenez et al. [6] reported that one-touch conditions elicited a greater frequency of high-intensity movements and accelerations compared to two-touch or unrestricted play, likely due to the increased tempo and reduced time available for decision-making. Similarly, Praca et al. [20] observed a reduction in physical effort when an additional player was introduced into the game, resulting in lower volumes of high-intensity running and fewer accelerations. This effect may be explained by the failure to proportionally adjust pitch dimensions with the increased number of players, thereby reducing the relative ApP. Such conditions can lead to player congestion, limiting space for movement and, consequently, reducing locomotor demands [15].

Within this context, the findings of the present study provide important practical implications for training design. Despite being predominantly used for tactical purposes, Large SSGs (10v10) were associated with greater high-intensity running distances and higher top-end speeds than the other formats investigated. These characteristics may make them useful when coaches aim to expose players to higher-speed locomotor demands, although direct comparisons with competitive match-play demands were beyond the scope of the present study. In contrast, in the present study, medium SSGs were associated with lower high-intensity running outputs than the largest formats, and may therefore be better suited for recovery-focused sessions, tactical refinement, or planned deloading periods. Conversely, small SSGs (5v5), typically performed on reduced pitch sizes with fewer players, were associated with frequent high-tempo actions such as short sprints, accelerations, and increased actions per minute. These formats also facilitate enhanced cognitive engagement, requiring quicker decision-making and technical execution under pressure, as reflected by the higher number of actions performed per minute [11].

Overall, these findings underscore the importance of aligning SSG design with specific training objectives. Coaches should carefully manipulate variables such as pitch size, player numbers, and task constraints to elicit the desired physiological, physical, and cognitive responses, ensuring that training stimuli are appropriately targeted across different phases of the training cycle. It is important to interpret these findings within the context of the study design. Because player numbers, ApP, pitch dimensions, goalkeeper inclusion, tactical objectives, and game rules varied simultaneously across the SSG formats, the observed differences cannot be attributed to any single design characteristic. Rather, they reflect the combined influence of the training configurations routinely implemented by the coaching staff within a professional soccer environment.

### 4.1. Practical Applications

Based on the findings of the present study, it is suggested that both small and large SSGs demonstrate distinct physical demands, particularly in relation to total running distance and movement intensity. When appropriately integrated, these formats may contribute to the optimal development of players’ physical capacity and overall match performance. However, several practical considerations should be taken into account when designing training interventions.

Notably, small SSGs, characterized by fewer players and reduced pitch dimensions, appear to limit players’ ability to reach and sustain high-speed running. Consequently, these formats may not provide sufficient stimulus for the development of maximal speed and high-intensity running capacity. In contrast, exposure to maximum running speeds was associated with large SSGs conducted on larger pitches, where greater space allows for the execution of high-speed and sprinting actions.

Accordingly, an overreliance on small SSGs may be insufficient on their own with the principle of specificity, particularly when the objective is to replicate or prepare players for the high-intensity demands of match-play. While small SSGs are advantageous for enhancing technical involvement such as increased ball contacts, decision-making frequency, and short, explosive movements they should be complemented with larger formats to ensure a well-rounded physical stimulus. Therefore, these findings support selecting SSG formats according to the intended objectives of the training session while recognizing that multiple task constraints contribute to the physical responses observed.

### 4.2. Limitations

First, the study was conducted within a single South African Premier Soccer League team, which may limit the generalisability of the findings to other teams, competitive levels, or playing styles. Second, the observational design did not allow for the standardisation of important task-configuration variables. The different SSG formats varied not only in player numbers but also in pitch dimensions, area per player, tactical objectives, the presence or absence of goalkeepers, goal size, supporting players, and game rules. Consequently, it is not possible to attribute the observed differences exclusively to player numbers or SSG size.

Third, the analysed SSGs were extracted from routine training sessions, where coaches prescribed different formats according to the objectives of the training week. Smaller SSGs were predominantly used during conditioning sessions, whereas larger formats were generally implemented during tactical sessions. These contextual differences may have influenced the physical responses observed.

Finally, positional differences were not examined in the present analysis. Given the known variation in physical demands between playing positions, future research should investigate how different SSG formats affect positional groups independently. Future studies should seek to standardise pitch dimensions, area per player, game rules, and coaching objectives to better isolate the influence of SSG format on player performance. Furthermore, research involving multiple professional teams and the inclusion of both internal and external load measures would enhance the ecological validity and applicability of the findings.

## 5. Conclusions

The present study investigated the physical demands associated with different small-sided game formats used by a South African Premier Soccer League team during the in-season period. Significant differences were observed across SSG formats for total distance covered, high-intensity running distance, power plays, top-end speed, and relative distance covered. Small-sided games (5v5) were associated with greater relative work rates and higher movement demands per minute, indicating that these formats were associated with greater relative work rates. In contrast, larger formats, particularly 10v10, were characterized by greater high-intensity running distances and higher top-end speeds, suggesting a greater capacity to expose players to match-relevant locomotor demands.

These findings suggest that different SSG formats may elicit distinct physical responses and should therefore be selected according to the specific objectives of a training session. Smaller formats may be appropriate when the aim is to increase player involvement and relative workload, whereas larger formats appear more suitable for exposing players to high-speed running and sprint-related activities which resemble locomotor characteristics typically reported during competitive match-play. Consequently, coaches should strategically manipulate player numbers, pitch dimensions, and task constraints to achieve specific physical training outcomes while maintaining the technical and tactical benefits associated with small-sided games.

## Figures and Tables

**Table 1:** Number of players and field dimensions.

Size	Number of Players	Number of Observations	Field Dimensions	Area per Player (m^2^·Player^−1^)	Game Duration (min)
Small	5v5	32	30 × 20	60 m^2^	10
Medium	6v6	29	30 × 30	75 m^2^	10
	7v7	32	40 × 40	114.2 m^2^	10
Large	8v8	33	80 × 50	250 m^2^	10
	9v9	28	80 × 50	222.2 m^2^	10
	10v10	34	110 × 65	357.5 m^2^	10

**Table 2:** Mean ± SD for the physical performance data of different small-sided games.

Size		Physical Performance for Different Small-Sided Games					
	*N*	Total Distance (km)	High-Intensity Running (m)	Power Plays	Top-End Speed (km·h^−1^)	Distance per Minute (m·min^−1^)	*p*-Value
*small*							
**5v5**	32	1.2 ± 0.1 ^bce^	113.9 ± 30.3 ^bc^	11.6 ± 2.5 ^bc^	24.4 ± 1.2 ^c^	126.2 ± 8.2 ^cef^	*p* < 0.001
*medium*							
**6v6**	29	1.1 ± 0.1	77.1 ± 27.0	8.1 ± 2.0	23.3 ± 1.5	119.8 ± 10.5 ^e^	*p* < 0.001
**7v7**	32	1.1 ± 0.1	79.0 ± 21.6	9.5 ± 2.3	23.4 ± 1.3	115.5 ± 9.6	*p* < 0.001
*large*							
**8v8**	33	1.2 ± 0.1 ^bce^	113.7 ± 37.5 ^bc^	11.1 ± 2.8 ^b^	24.4 ± 2.1 ^bc^	123.6 ± 11.0 ^e^	*p* < 0.001
**9v9**	28	1.1 ± 0.1	112.7 ± 33.9 ^bc^	9.7 ± 2.2	25.7 ± 1.5 ^abc^	108.3 ± 10.0	*p* < 0.001
**10v10**	34	1.2 ± 1.0 ^bce^	144.9 ± 38.3 ^abcde^	11.6 ± 2.3 ^bce^	26.3 ± 1.3 ^abcd^	119.8 ± 8.7 ^e^	*p* < 0.001

Data are presented as means ± SD. The level of significance was set at *p* < 0.05. Differences between SSGs indicated using letters (a, b, c, d, e & f) as follows: ^a^ denotes greater than 5v5; ^b^ greater than 6v6; ^c^ greater than 7v7; ^d^ greater than 8v8; ^e^ greater than 9v9; ^f^ greater than 10v10.

**Table 3:** Mean ± SD for the overall physical performance data per difference sizes.

Size		Overall Physical Performance per Different Sizes					
	*N*	Total Distance (km)	High-Intensity Running (m)	Power Plays	Top End-Speed (km·h^−1^)	Distance per Minute (m·min^−1^)	*p*-Value
small	32	1.2 ± 0.02 ^bc^	113.9 ± 5.3 ^b^	11.6 ± 0.4 ^b^	24.4 ± 0.2 ^b^	126.2 ± 1.4 ^bc^	*p* < 0.001
medium	61	1.1 ± 0.01	79.5 ± 3.1	8.9 ± 0.3	23.4 ± 0.2	117.1 ± 1.4	*p* < 0.001
large	95	1.2 ± 0.17 ^b^	122.4 ± 5.1 ^b^	10.8 ± 0.3 ^b^	25.4 ± 0.2 ^ab^	117.3 ± 1.5	*p* < 0.001

Data are presented as means ± SD. The level of significance was set at *p* < 0.05. Differences between SSG game sizes are indicated using letters (a, b & c) as follows: ^a^ denotes greater than small; ^b^ greater than medium; ^c^ greater than large.

## Data Availability

The data presented in this study are available on request from the corresponding author.
